# Validation of population‐level HIV‐1 incidence estimation by cross‐sectional incidence assays in the HPTN 071 (PopART) trial

**DOI:** 10.1002/jia2.25830

**Published:** 2021-12-12

**Authors:** Ethan Klock, Ethan Wilson, Reinaldo E. Fernandez, Estelle Piwowar‐Manning, Ayana Moore, Barry Kosloff, Justin Bwalya, Nomtha Bell‐Mandla, Anelet James, Helen Ayles, Peter Bock, Deborah Donnell, Sarah Fidler, Richard Hayes, Susan H. Eshleman, Oliver Laeyendecker, the HPTN071 (PopART) study team

**Affiliations:** ^1^ Division of Infectious Diseases Department of Medicine School of Medicine Johns Hopkins University Baltimore Maryland USA; ^2^ Fred Hutchinson Cancer Research Center Seattle Washington USA; ^3^ FHI 360 Durham North Carolina USA; ^4^ Zambart Lusaka Zambia; ^5^ Clinical Research Department London School of Hygiene and Tropical Medicine London UK; ^6^ Desmond Tutu Tuberculosis Center Department of Pediatrics and Child Health Faculty of Medicine and Health Sciences Stellenbosch University Cape Town South Africa; ^7^ Imperial College London London UK; ^8^ Department of Infectious Disease Epidemiology London School of Hygiene and Tropical Medicine London UK; ^9^ Department of Pathology School of Medicine Johns Hopkins University Baltimore Maryland USA; ^10^ National Institute of Allergy and Infectious Diseases National Institutes of Medicine Bethesda Maryland USA

**Keywords:** validation study, cross‐sectional incidence estimation, sub‐Saharan Africa, HPTN, PopART, multi‐assay algorithm

## Abstract

**Introduction:**

Cross‐sectional incidence testing is used to estimate population‐level HIV incidence and measure the impact of prevention interventions. There are limited data evaluating the accuracy of estimates in settings where antiretroviral therapy coverage and levels of viral suppression are high. Understanding cross‐sectional incidence estimates in these settings is important as viral suppression can lead to false recent test results. We compared the accuracy of multi‐assay algorithms (MAA) for incidence estimation to that observed in the community‐randomized HPTN 071 (PopART) trial, where the majority of participants with HIV infection were virally suppressed.

**Methods:**

HIV incidence was assessed during the second year of the study, and included only individuals who were tested for HIV at visits 1 and 2 years after the start of the study (2016–2017). Incidence estimates from three MAAs were compared to the observed incidence between years 1 and 2 (MAA‐C: LAg‐Avidity <2.8 ODn + BioRad Avidity Index <95% + VL >400 copies/ml; LAg+VL MAA: LAg‐Avidity <1.5 ODn + VL >1000 copies/ml; Rapid+VL MAA: Asanté recent rapid result + VL >1000 copies/ml). The mean duration of recent infection (MDRI) used for the three MAAs was 248, 130 and 180 days, respectively.

**Results and discussion:**

The study consisted of: 15,845 HIV‐negative individuals; 4406 HIV positive at both visits; and 221 who seroconverted between visits. Viral load (VL) data were available for all HIV‐positive participants at the 2‐year visit. Sixty four (29%) of the seroconverters and 3227 (72%) prevelant positive participants were virally supressed (<400 copies/ml). Observed HIV incidence was 1.34% (95% CI: 1.17–1.53). Estimates of incidence were similar to observed incidence for MAA‐C, 1.26% (95% CI: 1.02–1.51) and the LAg+VL MAA, 1.29 (95% CI: 0.97–1.62). Incidence estimated by the Rapid+VL MAA was significantly lower than observed incidence (0.92%, 95% CI: 0.69–1.15, *p*<0.01).

**Conclusions:**

MAA‐C and the LAg+VL MAA provided accurate point estimates of incidence in this cohort with high levels of viral suppression. The Rapid+VL significantly underestimated incidence, suggesting that the MDRI recommended by the manufacturer is too long or the assay is not accurately detecting enough recent infections.

## 1 INTRODUCTION

HIV incidence is the rate of new HIV infections in a population and the primary measure in evaluating the state of the epidemic and the effectiveness of prevention interventions [1]. Cross‐sectional incidence assays are used in multi‐assay algorithms (MAAs) to estimate HIV incidence [2, 3]. This approach is easier and more cost‐effective than estimating incidence using longitudinal cohort studies [2, 3]. Currently, the industry standard and most widely used testing algorithm for incidence estimation, including the Population Health Indicators Assessment (PHIA) and the Tracking with Recency Assays to Control the Epidemic (TRACE), is Limiting Antigen Avidity Assay plus viral load (LAg+VL) [4, 5, 6, 7, 8].

The accuracy of cross‐sectional incidence assays is influenced by several factors, most notably antiretroviral therapy (ART) [10, 11, 12]. ART suppresses viral replication, which in turn reduces the antibody response to HIV, which can increase the number of false recent results [11, 12, 13]. HIV viral load (VL) is often included in MAAs as a surrogate marker for non‐recent infection [14, 15], based on the assumption that the most individuals are not on treatment early in infection. The accuracy of cross‐sectional incidence estimates has not been assessed in populations with universal ART (i.e. ART initiated at any time).

We evaluated the performance of MAAs in a community‐randomized trial that evaluated the impact of a combination prevention package, including universal testing and treatment on HIV incidence: the HIV Prevention Trials Network (HPTN) 071 (PopART) trial. HIV incidence estimates from MAAs were compared to observed HIV incidence based on annual HIV testing. Three MAAs were evaluated: an MAA optimized for incidence estimation in subtype C epidemics (MAA‐C); the widely used MAA that includes the LAg assay plus VL (LAg+VL); and an MAA that includes a point‐of‐care assay [the Asanté HIV‐1 Rapid Recency Test plus VL (Rapid+VL)], currently being used in the TRACE program [2, 16]. Incidence estimates obtained with these MAAs were compared to observed incidence during longitudinal follow‐up.

## 2 METHODS

### 2.1 Study population

The sample set evaluated originated from the HPTN 071 trial (conducted between 2013 and 2018) in 21 communities in Zambia and South Africa [17]. Participants provided informed consent prior to study enrolment. No new specimens were obtained for this study. Research was conducted in accordance with the Declaration of Helsinki.

The HPTN 071 trial enrolled a randomly selected Population Cohort (PC) of 48,302 adults (age 18–44), who were followed for up to 3 years with four annual surveys (PC0, PC12, PC24 and PC36) [17]. Participants in the HPTN 071 trial lived in one of 21 communities located in Zambia and South Africa [18]. In total, 4627 HIV‐positive individuals were tested; 3500 who enrolled HIV+ at PC0, 689 who enrolled HIV+ at PC12, 217 who seroconverted between PC0 and PC12, and 221 who seroconverted between PC12 and PC24. We assessed HIV incidence between the first and second year of the trial (PC12 and PC24 study visits, 2016–2017) for 20,472 participants who had HIV status determined at both of these visits (Table 1).

**Table 1:** Participant demographics

	SC	HIV+	HIV–	Follow‐up (years)
Overall	221	4627	15,845	16,463
Study arm				
A	76	1507	5217	6724
B	60	1616	5918	7534
C	85	1504	4710	6214
Country				
South Africa	89	1753	6845	7105
Zambia	132	2874	9000	9358
Sex				
Female	190	4048	11,106	11,575
Male	31	619	4739	4888
18–24 years old				
Female	77	484	3441	3605
Male	11	50	1893	1951

Note: The table shows demographic characteristics of participants included in the study. Sex was assessed for all participants and for the subset of participants aged 18–24.

Abbreviations: Follow‐up, time between visits for uninfected participants and seroconverters, measured in years; HIV+, number of HIV seropositive individuals; HIV–, number of HIV seronegative individuals; SC, seroconverters.

### 2.2 Laboratory testing

Plasma samples from participants who were HIV positive at PC24 were tested using the HIV‐1 LAg‐Avidity EIA (LAg, Sedia Biosciences Corporation, Portland, OR, USA) and the Johns Hopkins modified BioRad‐Avidity assay [14, 19]. The LAg assay was performed using the manufacturer's protocol; results from this assay were reported as a normalized optical density (ODn). Results from the JHU‐BioRad‐Avidity assay were reported as Avidity Index (AI). The Asanté HIV‐1 Rapid Recency Assay (Rapid, Sedia BioSciences Corporation) was performed using the manufacturer's protocol; each test result was evaluated by the same technician for consistency. Testing with the Rapid assay was performed on all 221 SC and HIV‐positive participants who had a VL >1000 copies/ml. Additionally, a hundred samples from individuals known to be infected >2 years, with VLs <1000 copies/ml, were tested for validation purposes.

### 2.3 Data analysis

Incidence estimates from the three MAAs were compared to observed HIV incidence. Observational incidence was measured by dividing the number of observed seroconversions over the number of years of follow up for participants who were HIV‐uninfected at the first visit (PC12). HIV incidence is expressed as the number of infections per 100 person years. MAA‐C classifies infections as recent if they have a LAg result <2.8 ODn, an AI <95% and a VL >400 copies/ml. The mean duration of recent infection (MDRI) for this MAA is 248 days [14]. The LAg MAA (LAg+VL) classifies infections as recent if they have a LAg result <1.5 ODn and a VL >1000 copies/ml. The MDRI for this MAA is 130 days [20]. The Rapid MAA (Rapid+VL) classifies infections as recent if they have a Rapid result of “recent” and a VL >1000; the manufacturer of the Rapid assay indicates that this MAA has an MDRI of 180 days [21]. A false recent rate (FRR), defined as the proportion of individuals infected >2 years who were misclassified as recently infected, was set at 0% for all algorithms; the FRR indicates the percent of infections of >2 years duration that are misclassified as recent. Though the incidence estimates could be adjusted for a local FRR, we set the value at 0%, as that is what is currently used in the PHIA and other large African surveys [5, 6, 7, 8, 9]. Incidence estimates, confidence intervals (CIs) and differences in incidence were generated with CEPHIA's ABIE v3 Incidence Calculator [22]. Analyses were also conducted in defined sub‐populations to assess the performance of the three MAAs in different participant groups, defined by study arm, country, sex and sex among young people.

## 3 RESULTS AND DISCUSSION

The analysis included: 221 individuals who acquired HIV infection between PC12 and PC24 (PC12–24 seroconverters, SC); 15,845 individuals who were HIV negative at PC12 and PC24; 4189 participants who were HIV positive at enrolment (3500 enrolled at PC0; 689 enrolled at PC12); and 217 individuals who were negative at PC0, but positive at PC12 and PC24. VL data were obtained in the parent study [12]. Seventy‐three percent (3390/4627) of all HIV‐positive individuals were virally suppressed (VL <400 copies/ml) at the PC24 visit; this included 29% (64/221) of the seroconverters included in this study (SC group).

**Figure 1:**
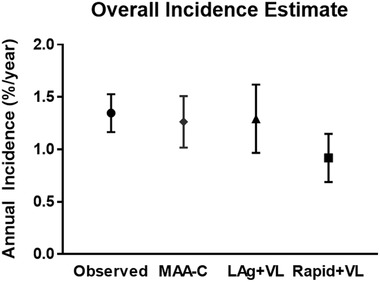
Comparison of cross‐sectional incidence estimates to observed incidence in HPTN 071 (PopART). The figure shows a comparison of annual HIV incidence observed in the HPTN 071 (PopART) trial, and incidence estimated with three multi‐assay algorithms (MAAs). Abbreviations: LAg, limiting antigen avidity assay; MAA‐C, Clade C multi‐assay algorithm; Rapid, Asante rapid LAg test assay; VL, viral load.

In this study, the observed HIV incidence between the PC12 and PC24 surveys was 1.34 (95% CI: 1.17–1.53). Of the 4627 HIV‐positive participants, 1295 had a LAg result <2.8 ODn, 1508 had a JHU‐BioRad‐Avidity assay AI <95% and 1337 had a VL >400 copies/ml; 136 participants met all three criteria and were classified as recent by MAA‐C. One‐hundred of these 136 individuals were known to be infected <1 year and 18 of 3500 (0.5%) were known to be infected >2 years. For the LAg+VL MAA, 535 had LAg result <1.5 ODn and 1234 had a VL >1000 copies/ml; 73 participants met both of these criteria and were classified as recent by this MAA. Sixty of these 73 individuals were known to be infected <1 year and 6 of 3500 (0.2%) were known to be infected >2 years. For the Rapid+VL MAA, 1234 participants had a VL >1000 copies/ml and 72 did not have a detectable long‐term band; these 72 participants were classified as recent. Fifty‐three of these 72 participants were known to be infected <1 year and 16 of 3500 (0.5%) were known to be infected >2 years. The incidence estimates for the MAA‐C, LAg+VL and Rapid+VL MAAs were 1.26 [95% CI: 1.02–1.51], 1.29 [95% CI: 0.97–1.62] and 0.92 [95% CI: 0.69–1.15], respectively (Figure 1).

**Figure 2:**
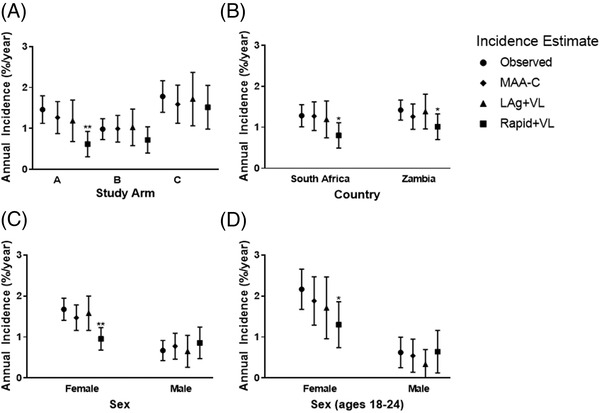
Comparison of cross‐sectional incidence estimates to observed incidence in HPTN 071 (PopART) by study arm, country, sex and sex among young persons. The plots show observed incidence and incidence estimates for three multi‐assay algorithms (MAAs). The circles represent observed incidence based on longitudinal follow‐up. The sub‐analyses are presented by study arm (a); country (b); sex (c); and sex among young (aged 18–24) individuals (d); 95% confidence intervals are shown for each point estimate of incidence. Incidence estimates that differ significantly from the observed incidence are noted (* *p*<0.05; ** *p*<0.01). Abbreviations: LAg, limiting antigen avidity assay; MAA‐C, Clade C optimized multi‐assay algorithm; Rapid, Asante rapid LAg test assay; VL, viral load.

Incidence estimates were also generated for participant sub‐groups, based on study arm, country, sex and sex in younger participants (aged 18–24) (Figure 2a–d). In these analyses, the MAA‐C and LAg+VL MAA incidence estimates were not significantly different from the observed incidence. In contrast, incidence estimates obtained using the Rapid+VL MAA were significantly lower than the observed incidence in many sub‐groups (for study arm A: 0.62 [95% CI: 0.31–0.93] vs. 1.42 [95% CI: 1.15–1.82], *p* = 0.001; for South Africa: 0.80 [95% CI: 0.49–1.11] vs. 1.27 [95% CI: 1.02–1.56], *p*<0.05; for Zambia, 1.01 [95% CI: 0.70–1.33] vs. 1.41 [95% CI: 1.18–1.67], *p*<0.05; for females: 0.95 [95% CI: 0.68–1.23] vs. 1.65 [95% CI: 1.42–1.96], *p*<0.001; for females aged 18–24, 1.30 [95% CI: 0.74–1.86] vs. 2.14 [95% CI: 1.69–2.67], *p*<0.05). All data analysed are available in the Supporting Information.

In the Rapid+VL algorithm, a total of 10 samples did not have a reactive HIV band. None of these individuals had reactive long‐term bands either. The median LAg value for these samples was 0.17 ODn, though four samples did have values above 0.5 ODn. Nine of 10 samples were from virally suppressed individuals. Among the 100 samples from individuals infected >2 years with VLs <1000 copies/ml, 32 did not have a visible long‐term infection band. Five of these samples were among those appearing to be HIV uninfected. All of these individuals were sero positive by the ARCHITECT HIV Ag/Ab COMBO test and Geenius HIV‐1/2 Supplemental assay [23].

Our study demonstrated that the MAA‐C and the LAg+VL MAA provided accurate point estimates of HIV incidence in a population with a high frequency of viral suppression. In a previous study, MAA‐C and LAg+VL MAA also provided accurate point estimates of incidence in a subtype C epidemic setting where ART was not widespread [24]. Although the point estimates of incidence were accurate for both assays, the MAA‐C estimates had smaller CIs than the LAg+VL MAA estimates, and the point estimates of incidence were closer to observed incidence for MAA‐C in most of the sub‐group analyses. MAA‐C also identified more individuals with recent infection and had incidence estimates with smaller CIs reflecting the longer MDRI of this algorithm.

The Rapid+VL MAA was the only MAA that yielded an incidence estimate that was consistently and significantly lower than observed incidence. This MAA significantly underestimated HIV incidence overall and in all sub‐groups except for male participants, the population with the lowest observed incidence. The poor performance of this MAA suggests that it may not be as effective as the other MAAs to identify clusters of recent infections. The Consortium for the Evaluation and Performance of HIV Incidence Assays (CEPHIA) estimated an MDRI of 83 days for this MAA in a non‐peer reviewed study [25]. The low incidence estimates obtained in our study support CEPHIA's findings, indicating that the MDRI for the Rapid+VL MAA could be lower than 180 days. The Rapid+VL algorithm identified almost the same number of recent infections as the LAg+VL algorithm (72 and 73, respectively), suggesting that an MDRI of approximately 130 days may be more appropriate. Future research is needed to determine the MDRI for this MAA in populations that include widespread ART.

Our study has limitations. Observed incidence was assessed over 1 year of follow‐up, while the incidence estimates were calculated using MAAs with window periods <1 year. However, the time periods assessed by the two methods are overlapping, and incidence was not likely to have changed significantly during the 1‐year period. The HPTN 071 cohort was conducted in Zambia and South Africa, where most infections are caused by HIV subtype C [26]. Further research is needed in populations with other prevalent subtypes. None of the MAAs evaluated were perfect in their ability to eliminate false recent misclassification. Among the samples classified as recently infected, 13% (18/136), 8% (6/73) and 22% (16/72) of MAA‐C, LAg+VL and Rapid+VL were known to be infected>2 years. Another limitation of our study is that we did not include testing for antiretroviral biomarkers. Future research should seek to determine if the addition of ART biomarker testing would significantly improve the results of one or all of these algorithms.

## 4 CONCLUSIONS

As ART becomes more widely available across the globe, and as universal ART is more widely implemented, it is especially important to identify MAAs that perform well in populations with high rates of early ART and viral suppression. Our study demonstrates that the MAA‐C and LAg+VL MAA can provide accurate incidence estimates in current universal treatment settings.

## COMPETING INTERESTS

The authors have no competing interests to declare.

## AUTHORS’ CONTRIBUTIONS

EK, DD, SHE and OL designed the study. AM, BK, JB, NB‐M, AJ, HA, PB and SF had primary responsibility for the blood specimen and data collection. EK, EP‐M and REF performed laboratory testing. EW, OL, BK, AJ and DD supervised data collection. OL, EP‐M, SHE and REF supervised laboratory testing. OL, EW and DD performed data analyses. EK, EW, SHE and OL interpreted primary results. EK, SHE and OL primarily drafted the manuscript. AM, BK, JB, NB‐M, AJ, HA, PB, DD, SF and RH performed critical editing of the manuscript. EK, EW, REF, EP‐M, AM, BK, JB, NB‐M, AJ, HA, PB, DD, SF, RH, SHE and OL reviewed and approved the manuscript.

## Data Availability

The data used in the manuscript is available in Supplemental File 1.
